# Survival and Symptomatic Relief After Cytoreductive Hepatectomy for Neuroendocrine Tumor Liver Metastases: Long-Term Follow-up Evaluation of More Than 500 Patients

**DOI:** 10.1245/s10434-023-13372-z

**Published:** 2023-05-19

**Authors:** Hallbera Gudmundsdottir, Elizabeth B. Habermann, Robert A. Vierkant, Patrick Starlinger, Cornelius A. Thiels, Susanne G. Warner, Rory L. Smoot, Mark J. Truty, Michael L. Kendrick, Thorvardur R. Halfdanarson, David M. Nagorney, Sean P. Cleary

**Affiliations:** 1 Department of Surgery, Mayo Clinic, Rochester, MN USA; 2 Division of Health Care Delivery Research, Robert D. and Patricia E. Kern Center for the Science of Health Care Delivery, Mayo Clinic, Rochester, MN USA; 3 Department of Quantitative Health Sciences, Mayo Clinic, Rochester, MN USA; 4 Department of Medical Oncology, Mayo Clinic, Rochester, MN USA

## Abstract

**Background:**

Distant metastases are the strongest predictor of poor prognosis for patients with neuroendocrine tumors (NETs). Cytoreductive hepatectomy (CRH) can relieve symptoms of hormonal excess and prolong survival for patients with liver metastases (NETLMs), but long-term outcomes are poorly characterized.

**Methods:**

This retrospective single-institution analysis analyzed patients who underwent CRH for well-differentiated NETLMs from 2000 to 2020. Kaplan-Meier analysis estimated symptom-free interval and overall and progression-free survival. Multivariable Cox regression analysis evaluated factors associated with survival.

**Results:**

The inclusion criteria were met by 546 patients. The most common primary sites were the small intestine (*n* = 279) and the pancreas (*n* = 194). Simultaneous primary tumor resection was performed for 60 % of the cases. Major hepatectomy comprised 27% of the cases, but this rate decreased during the study period (*p* < 0.001). Major complications occurred in 20%, and the 90-day mortality rate was 1.6%. Functional disease was present in 37 %, and symptomatic relief was achieved in 96%. The median symptom-free interval was 41 months (62 months after complete cytoreduction and 21 months with gross residual disease) (*p* = 0.021). The median overall survival was 122 months, and progression-free survival was 17 months. In the multivariable analysis, worse overall survival was associated with age, pancreatic primary tumor, Ki-67, number and size of lesions, and extrahepatic metastases, with Ki-67 as the strongest predictor (odds ratio [OR], 1.90 for Ki-67 [3–20%; *p* = 0.018] and OR, 4.25 for Ki-67 [>20%; *p* < 0.001]).

**Conclusion:**

The study showed that CRH for NETLMs is associated with low perioperative morbidity and mortality and excellent overall survival, although the majority will experience recurrence/progression. For patients with functional tumors, CRH can provide durable symptomatic relief.

Neuroendocrine tumors (NETs) are rare and heterogeneous tumors that most commonly arise in the gastrointestinal tract, pancreas, and lung.^1^ Although they generally exhibit more indolent behavior than typical epithelial malignancies, up to 60% of patients have distant metastases at the time of diagnosis.^2^ Distant metastases, the strongest predictor of a poor prognosis, in about 90% of cases are located in the liver.^3–5^ In a study using the Surveillance, Epidemiology, and End Results (SEER) database, the median survival time from diagnosis was 65 months for patients with metastatic small intestinal NETs and 27 months for patients with metastatic pancreatic NETs.^2^

Cytoreductive hepatectomy for NET liver metastases was initially described in 1977 as a way to control symptoms of hormonal excess in patients with carcinoid syndrome or functional islet cell tumors.^6,7^ Indications subsequently expanded to include nonfunctional tumors after several retrospective studies from our institution, and other studies showed better survival after hepatectomy than after nonoperative therapy and historical controls.^7–13^ Although rarely curative, cytoreductive hepatectomy is believed to prolong survival by “setting back the clock” and delaying the development of liver failure caused by hepatic tumor replacement, the leading cause of death among patients with metastatic NETs.^14^

The use of cytoreductive hepatectomy for patients with nonfunctional tumors remains somewhat controversial due to the retrospective nature of the supporting evidence, but prospective randomized trials are difficult to conduct with this population due to the rarity of the disease and the relatively indolent disease course necessitating a long follow-up period. Currently, consensus guidelines by the North American (NANETS) and European (ENETS) Neuroendocrine Tumor Societies recommend that treatment be individualized with consideration of hepatectomy when anatomically feasible, but it remains unknown which patients benefit the most from operative management.^14–16^

Our group has previously described the outcomes for patients who underwent cytoreductive hepatectomy for NET liver metastases in the pre-2000 era.^9^ In this report, we provide a modern-day update, evaluating perioperative outcomes and operative trends over time, rates and duration of symptomatic relief, and long-term survival and predictors of prognosis through a review of one of the largest institutional experiences to date.

## Methods

### Study cohort and data collection

This study was approved by the Mayo Clinic Institutional Review Board. Patients who underwent hepatectomy for metastatic well-differentiated gastroenteropancreatic, bronchopulmonary, or genitourinary NETs from January 2000 to December 2020 were identified, and relevant data were obtained were from medical records. Based on case numbers, primary tumor sites were grouped into small intestinal NETs (not including duodenum), pancreatic NETs, and NETs of all other primary sites. For patients who underwent more than one hepatic resection, only the first procedure was included. Patients who had undergone a prior hepatectomy before 2000 or at an outside facility were similarly excluded.

Cytoreduction in all patients consisted of resection with or without concurrent intraoperative ablation. Extent of cytoreduction was estimated using operative notes (including descriptions of intraoperative ultrasound) and by comparing pre- and postoperative cross-sectional imaging. The extent of cytoreduction was categorized as complete cytoreduction (with all visible lesions addressed), incomplete (with >90% cytoreduction), and incomplete (with <90% cytoreduction). Expression of Ki-67 was determined by immunohistochemical staining and reported as the percentage of evaluated tumor cells that stained positive for Ki-67.

Major hepatectomy was defined as right hepatectomy or trisegmentectomy (with or without minor resection/ablation of the contralateral lobe) and minor hepatectomy (all other types of resection) based on morbidity and mortality data from the National Surgical Quality Improvement Program (NSQIP) validated for our cohort.^17^ The Clavien-Dindo system was used to classify postoperative complications occurring within 90 days after surgery and major complications defined as Clavien-Dindo 3 or greater.^18^ Hepatectomy-specific complications such as posthepatectomy bile leakage (PHBL), hemorrhage (PHH), and liver failure (PHLF) were defined and graded according to the respective International Study Group of Liver Surgery (ISGLS) classifications.^19–21^ Progression was defined as radiographic evidence of recurrence or increased tumor burden with or without pathologic confirmation.

Patients were considered to have functional disease if they had symptoms consistent with carcinoid syndrome or functional islet cell tumor documented before surgery by the treating medical and/or surgical oncologist. All patients with carcinoid heart disease had the diagnosis confirmed with preoperative echocardiogram. Patients who died within 90 days after surgery or were immediately lost to follow-up assessment were excluded from evaluation of symptomatic relief. The presence or absence of symptoms showing hormonal excess was documented by the treating medical and/or surgical oncologist at the postoperative follow-up evaluation of all the patients. Symptomatic relief was defined as patient-reported resolution or a marked improvement in symptoms at the first postoperative clinic visit. If symptoms were well controlled with medications before surgery, patients were considered to have symptomatic relief if a stable dose was able to be reduced or medications were discontinued altogether.

### Statistical Analysis

All statistical calculations were performed using R (version 4.0.0). Two-sided *p* values lower than 0.05 were considered statistically significant. Continuous variables are reported as median and interquartile range (IQR) and categorical variables as number and percentage. In univariate analysis, chi-square or Fisher’s exact tests were used to compare categorical variables, and Wilcoxon rank-sum test or Kruskal–Wallis one-way ranked analysis of variance was used to compare the medians of continuous variables. Trends over time were evaluated using the Cochran–Armitage test.

Overall survival, progression-free survival, and symptom-free interval after hepatectomy were estimated according to the Kaplan-Meier method, and differences observed among patient subgroups were assessed using the log-rank test. Time-to-event outcomes were calculated from the date of hepatectomy to the date of death, radiographic progression, or patient-reported return of symptoms, or to the date of the last follow-up visit for patients who did not have an event. For symptom-free interval, patients were additionally censored if they continued or started to receive somatostatin analogs (SSAs) for antiproliferative purposes in the absence of symptoms to avoid falsely prolonging the symptom-free interval. Similarly, in gastrinoma cases only, patients were censored if they continued or started to receive acid-reducing medications for preventive purposes in the absence of symptoms.

At our institution, Ki-67 rarely was reported before the publication of the 2010 World Health Organization (WHO) classification for gastrointestinal NETs. Multiple imputation was used to fill in missing values of Ki-67 using the R package MICE*.*^22^ To estimate continuously distributed Ki-67 levels, 40 imputed data sets were generated using the following variables: age at hepatectomy, biologic sex, functional tumor status, primary tumor site, number of metastases, size of largest liver metastasis, presence of extrahepatic distant metastases, overall survival time and status, and progression-free survival time and status.

After imputation, Ki-67 values were categorized into three groups (< 3%, 3–20%, and > 20%) for each imputed data set. Cox regression hazard ratios, confidence intervals, and *p* values summarized across the imputed data sets were calculated using Rubin’s rules.^23,24^ Summary Ki-67 survival curves were generated by fitting a survival curve to each of the 40 imputed data sets, extracting survival probabilities at half-year increments after hepatectomy, transforming survival probabilities to hazard ratios using properties of the exponential distribution, averaging the time-specific hazard ratios across the 40 data sets, transforming these average hazard ratios back to the survival scale, and plotting the survival values using a loess smoother. For the summary Ki-67 survival curves, the number of patients at risk at each time point was estimated by averaging the individual numbers at risk for each imputed dataset.

## Results

### Study Cohort

From 2000 to 2020, 546 patients underwent their first cytoreductive hepatectomy for NET liver metastases at Mayo Clinic Rochester. The most common primary tumor site was the small intestine (*n* = 279, 51%), followed by the pancreas (*n* = 194, 36%). The remaining patients (*n* = 73, 13%) had tumors of other gastroenteric (*n* = 27), bronchopulmonary (*n* = 10), or genitourinary (*n* = 7) origin or had an unknown primary tumor site (*n* = 29).

Patient characteristics are shown in Table 1. The median age was 59 years, and 49% of the patients were female. Liver metastases were synchronous in 77% and metachronous in the 23% of the patients. Tumors were functional in 37% of the patients. For 235 of the patients (43%), Ki-67 was available and functioned less than 3% in 29%, 3% to 20% in 53%, and more than 20% in 17% of the patients. The median size of the largest liver lesion was 42 mm, and 52% of the patients had 10 or more lesions. Extrahepatic distant metastases were present in 16% of the patients. The most common sites of extrahepatic distant metastases were the peritoneum (*n* = 60), bone (*n* = 28), and lung (*n* = 3). Complete cytoreduction was achieved in 75%, incomplete (> 90%) cytoreduction in 20%, and incomplete (< 90%) cytoreduction in 4.8% of the patients. The patients with small intestinal tumors were older (median 61 vs. 57 and 55 years; *p* < 0.001), had a lower Ki-67 index (10% of values >20% vs. 24%, and 26%; *p* < 0.001), and were more likely to have functional tumors (53% vs. 17% and 26%; *p* < 0.001) than those with tumors from other sites. The patients with small intestinal NETs had the most numerous liver lesions (≥ 10 lesions in 58% vs. 48% and 38%; *p* = 0.030) and were more likely to have extrahepatic distant metastases (24% vs. 7.7% and 9.6%; *p* < 0.001), whereas those with primary tumor sites other than the small intestine and pancreas had the largest lesions (median, 58 vs. 40 and 38 mm; *p* = 0.028). Extent of cytoreduction was similar between the primary tumor site groups (*p* = 0.95). The prevalence of extrahepatic distant metastases increased significantly during the study period (*p* = 0.009), from 12% in the first third to 22% in the last third.

**Table 1:** Characteristics of patients who underwent hepatectomy for neuroendocrine tumor liver metastases^a^

	All patients (*n* = 546)	Small intestine (*n* = 279)	Pancreas (*n* = 194)	Other sites (*n* = 73)	*p* Value
	*n* (%)	*n* (%)	*n* (%)	*n* (%)	
Median age: years (IQR)	59 (50–66)	61 (53–69)	57 (48–64)	55 (48–65)	< 0.001
Sex					
Male	280 (51)	143 (51)	104 (54)	33 (45)	0.47
Female	266 (49)	136 (49)	90 (46)	40 (55)	
Functional tumor					
No	345 (63)	130 (47)	161 (83)	54 (74)	< 0.001
Yes	201 (37)	149 (53)	33 (17)	19 (26)	
Ki-67 index (%)^b^					
< 3	69 (29)	50 (44)	12 (13)	7 (26)	< 0.001
3–20	125 (53)	53 (46)	59 (63)	13 (48)	
> 20	41 (17)	12 (10)	22 (24)	7 (26)	
No. of liver lesions					
1–3	138 (25)	62 (22)	51 (26)	25 (34)	0.030
4–9	126 (23)	56 (20)	50 (26)	20 (27)	
≥ 10	282 (52)	161 (58)	93 (48)	28 (38)	
Size of largest lesion					
Median: mm (IQR)	42 (21–70)	40 (20–70)	38 (20–65)	58 (30–87)	0.028
Extrahepatic distant metastases					
No	456 (84)	211 (76)	179 (92)	66 (90)	< 0.001
Yes	90 (16)	68 (24)	15 (7.7)	7 (9.6)	
Extent of cytoreduction					
Complete cytoreduction	410 (75)	209 (75)	146 (75)	55 (75)	0.95
Incomplete debulking (> 90%)	110 (20)	56 (20)	38 (20)	16 (22)	
Incomplete debulking (< 90%)	26 (4.8)	14 (5.0)	10 (5.2)	2 (2.7)	

IQR, interquartile range

^a^Categorical variables are presented as number (%) and continuous variables as median (IQR)

^b^Patients with missing Ki-67 values (*n* = 307) were excluded from the respective univariate analysis

### Perioperative Outcomes

**Fig. 1:**
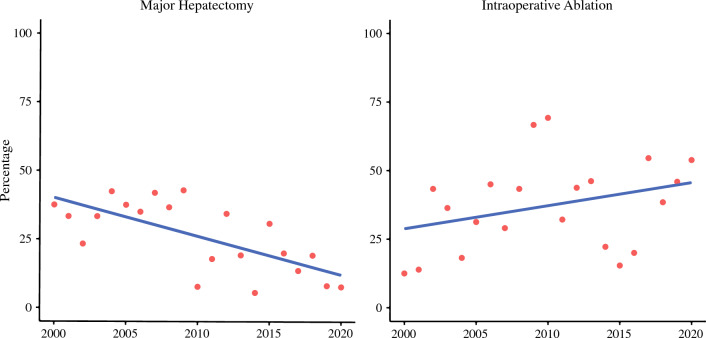
Trends in the use of major hepatectomy and intraoperative ablation over time**.** Data are shown as the percentage of all hepatectomies performed per year. The rate of major hepatectomy decreased during the study period (*p* < 0.001), whereas the use of intraoperative ablation increased (*p* < 0.001)

During the entire study period, major hepatectomy was performed for 146 patients (27%) and minor hepatectomy for 400 patients (73%). Intraoperative ablation was used in combination with resection in 205 cases (38%). The use of major hepatectomy decreased during the study period, whereas the use of intraoperative ablation increased (both *p* < 0.001) (Fig. 1). Simultaneous primary tumor resection was performed for 327 patients (60%) including 249 (62%) of 400 patients who underwent minor hepatectomy and 78 (54%) of 146 patients who underwent major hepatectomy (*p* = 0.08).

Perioperative outcomes after hepatectomy are shown in Table 2. The patients who underwent major hepatectomy had longer operative times (median 249 vs. 239 min; *p* = 0.033) and greater estimated blood loss (> 1000 ml in 24% vs. 16%; *p* = 0.041). They also had higher rates of any major complication (29% vs. 17%; *p* = 0.002) and hepatectomy-specific complications, such as grade B/C PHBL (14% vs. 3.8%; *p* < 0.001), grade B/C PHH (6.8% vs. 1.5%; *p* = 0.003), and grade B/C PHLF (6.2% vs. 0.0%; *p* < 0.001).

**Table 2:** Perioperative outcomes after hepatectomy^a^

	All patients	Minor hepatectomy	Major hepatectomy	*p* Value
	(*n* = 546)	(*n* = 400)	(*n* = 146)	
	*n* (%)	*n* (%)	*n* (%)	
Median operative time: min (IQR)	241 (191–302)	239 (186–300)	249 (208–319)	0.033
EBL > 1000 (ml)	91 (18)	58 (16)	33 (24)	0.041
Any major complication	108 (20)	66 (17)	42 (29)	0.002
Grade B/C PHBL	35 (6.4)	15 (3.8)	20 (14)	< 0.001
Grade B/C PHH	16 (2.9)	6 (1.5)	10 (6.8)	0.003
Grade B/C PHLF	9 (1.6)	0 (0.0)	9 (6.2)	< 0.001
Median hospital stay: days (IQR)	7 (5–9)	6 (5–8)	8 (6–13)	< 0.001
Unplanned readmission	56 (10)	42 (11)	14 (9.6)	0.88
Unplanned reoperation	35 (6.4)	18 (4.5)	17 (12)	0.005
90-Day mortality	9 (1.6)	4 (1.0)	5 (3.4)	0.06

IQR, interquartile range; EBL, estimated blood loss; PHBL, post-hepatectomy bile leak; PHH, post-hepatectomy hemorrhage; PHLF, post-hepatectomy liver failure

^a^Categorical variables are presented as number (percentage) and continuous variables as median (IQR)

^a^Patients with missing information on operative time (*n* = 4) and EBL (*n* = 40) were excluded from the respective univariate analyses

The median hospital of stay was longer after major hepatectomy (8 vs. 6 days; *p* < 0.001), and although readmission rates were similar (9.6% vs. 11%; *p* = 0.88), the patients who underwent major hepatectomy had higher rates of unplanned reoperation (12% vs. 4.5%; *p* = 0.005). The most common reasons for unplanned reoperation were bleeding (*n* = 10), bowel obstruction, ischemia, or perforation (*n* = 9), enteric anastomotic leak (*n* = 5), fascial dehiscence (*n* = 4), and bile leak/biliary injury (*n* = 3). The mortality rate at 90 days was 3.4% after major hepatectomy and 1.0 % after minor hepatectomy (*p* = 0.06). Across the entire cohort, the rates for major complications (*p* = 0.63) and 90-day mortality (*p* = 0.37) did not change during the study period.

### Symptomatic Relief

Of the 546 patients, 201 (37%) had functional tumors with symptoms of hormonal excess before hepatectomy. The most common syndrome was carcinoid syndrome (*n* = 161), followed by gastrinoma (*n* = 18) and insulinoma (*n* = 9). Of the patients with carcinoid syndrome, 38 (24%) had carcinoid heart disease and 27 (17%) underwent tricuspid and/or pulmonary valve replacement before hepatectomy.

After the exclusion of patients who died within 90 days after surgery or were immediately lost to follow-up evaluation, 178 patients were evaluated for symptomatic relief. After hepatectomy, 170 patients (96%) were rendered asymptomatic or reported a marked improvement in symptoms, whereas 8 patients (4.5%) reported minimal or no improvement. The rate of symptomatic relief was 96% for carcinoid syndrome, 94% for gastrinoma, 100% for insulinoma, and 82% for other syndromes (*p* = 0.14). By extent of cytoreduction, the rate of symptomatic relief was 95% for the patients who underwent resection of all gross disease and 96% for those who had gross residual disease (*p* = 0.99).

**Fig 2:**
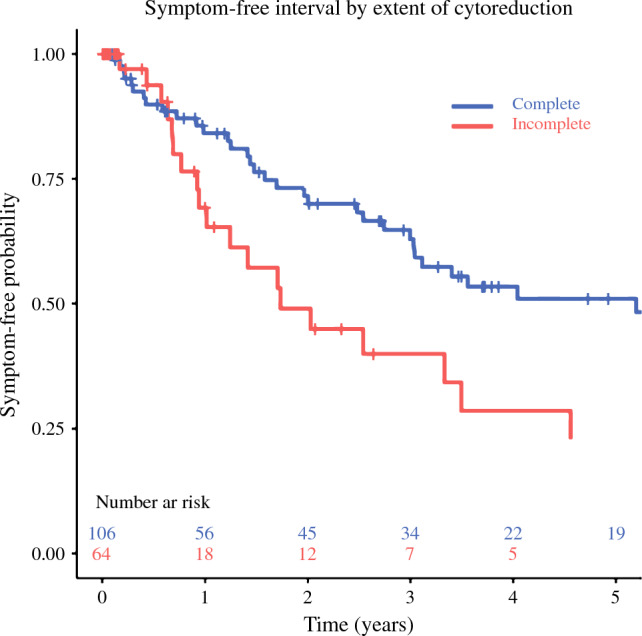
Symptom-free interval from hepatectomy for patients who experienced symptomatic relief stratified by extent of cytoreduction (*p* = 0.021). Curves are truncated when fewer than five patients remain at risk

Overall, the median symptom-free interval was 41 months (95 % confidence interval [CI], 30–71 months). Kaplan–Meier curves for symptom-free interval stratified by extent of cytoreduction are shown in Fig. 2 and differed significantly between groups (*p* = 0.021). The median symptom-free interval was 62 months (95% CI, 36–92 months) for the patients who had complete cytoreduction, compared with 21 months (95% CI, 12 to not reached) for the patients who had gross residual disease.

The median time from radiographic progression to return of symptoms was 8 months (95% CI, 2–24 months). Among the patients who had return of symptoms, 73% experienced symptoms within 1 year after radiographic progression.

### Overall and Progression-Free Survival

In the entire cohort, overall survival from hepatectomy was 75% (95% CI, 71–79%) at 5 years, 51% (95% CI, 46–57%) at 10 years, and 29% (95% CI, 23–36%) at 15 years. The median overall survival from hepatectomy was 122 months (95% CI, 109–142 months).

The progression-free survival from hepatectomy was 40% (95% CI, 36–45%) at 2 years and 19% (95% CI, 15–23%) at 5 years. The median progression-free survival from hepatectomy was 17 months (95% CI, 15–20 months).

**Fig. 3:**
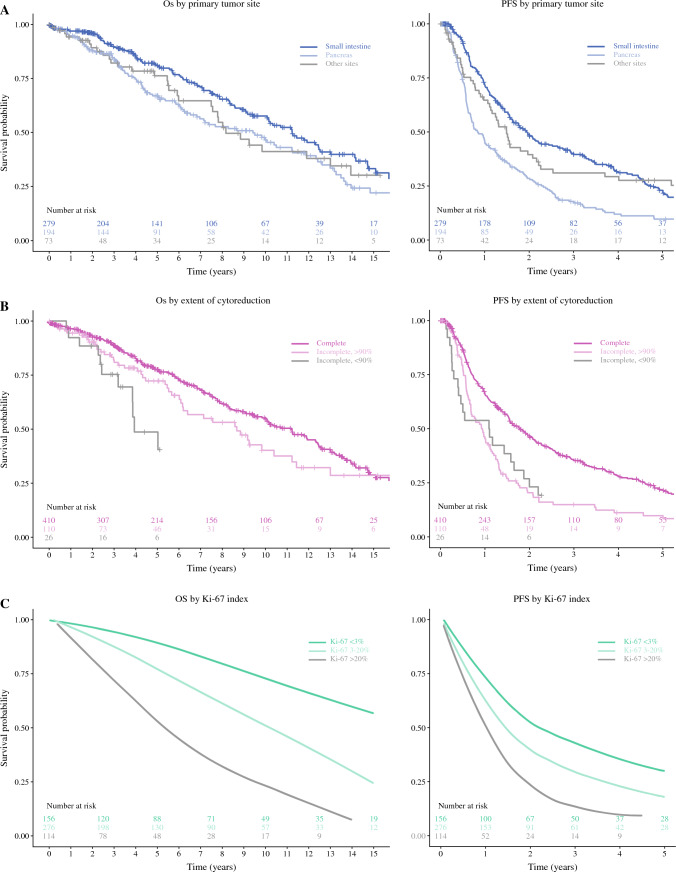
Overall survival (OS) and progression-free survival (PFS) from hepatectomy. **A** Stratification of OS (*p* = 0.038) and PFS (*p* < 0.001) by primary tumor site. **B** Stratification of OS (*p* = 0.06) and PFS (*p* < 0.001) by extent of cytoreduction. **C** Stratification of OS (*p* < 0.001) and PFS (*p* < 0.001) by Ki-67 index. A loess smoother was used to generate summary survival curves for each of the Ki-67 subgroups after multiple imputation. Survival curves are truncated when fewer than five patients remain at risk (**A–B**) or after the last documented event (**C**)

Kaplan–Meier curves for overall and progression-free survival stratified by primary tumor site, extent of cytoreduction, and Ki-67 are shown in Fig. 3. The overall survival curves differed significantly when stratified by primary tumor site (*p* = 0.038) and Ki-67 index (*p* < 0.001), but not when stratified by extent of cytoreduction (*p* = 0.06). The progression-free survival curves differed significantly when stratified by primary tumor site (*p* < 0.001), extent of cytoreduction (*p* < 0.001), and Ki-67 index (*p* < 0.001).

After division of the study period into 7-year periods (2000–2006; 2007–2013; 2014–2020), a statistically significant difference over time was found for overall survival (*p* = 0.017) but not for progression-free survival (*p* = 0.80). The 5-year overall survival rate was 71% (95% CI, 65–78%) in the first third, 78% (95% CI, 71–84%) in the second third, and 81% (95% CI, 73–89%) in the last third. The 10-year overall survival rate was 44% (95% CI, 37–53%) in the first third and 58% (95% CI, 51–67%) in the second third. Due to progression, a second cytoreductive hepatectomy was eventually performed for 41 patients (7.5%).

### Predictors of Overall and Progression-Free Survival

The results from the uni- and multivariable Cox proportional hazards regression analysis of factors associated with overall survival from hepatectomy are shown in Table 3. In the univariate analysis, worse overall survival was associated with advanced age, pancreatic primary tumor, higher Ki-67, greater number and size of liver lesions, extrahepatic distant metastases, and less than 90% debulking (all *p* < 0.05). In the multivariable analysis, worse overall survival remained associated with advanced age, pancreatic primary tumor, higher Ki-67, greater number and size of liver lesions, and extrahepatic distant metastases (all *p* < 0.05), whereas associations with extent of cytoreduction attenuated to nonsignificance.

**Table 3:** Uni- and multivariable Cox regression analysis of factors associated with overall survival from hepatectomy^a^

	Univariate analysis		Multivariable analysis	
	HR (95 % CI)	*p* Value	HR (95 % CI)	*p* Value
Age at hepatectomy				
Increase per year	1.02 (1.00–1.03)	0.013	1.02 (1.00–1.03)	0.009
Sex				
Male	1.00 (reference)			
Female	0.91 (0.70–1.18)	0.56		
Primary tumor site				
Small intestine	1.00 (reference)		1.00 (reference)	
Pancreas	1.43 (1.08–1.90)	0.012	1.49 (1.05–2.12)	0.026
All other sites	1.26 (0.85–1.88)	0.25	1.35 (0.85–2.16)	0.20
Functional tumor				
No	1.00 (reference)			
Yes	0.87 (0.66–1.14)	0.30		
Ki-67 index (%)				
< 3	1.00 (reference)		1.00 (reference)	
3–20	2.39 (1.27–4.51)	0.008	1.90 (1.12–3.24)	0.018
> 20	5.30 (2.58–10.90)	< 0.001	4.25 (2.35–97.66)	< 0.001
No. of metastases				
1–3 lesions	1.00 (reference)		1.00 (reference)	
4–9 lesions	1.56 (1.06–2.29)	0.023	1.58 (1.03–2.43)	0.046
≥ 10 lesions	1.50 (1.07–2.09)	0.019	1.32 (0.88–1.97)	0.18
Size of largest metastasis				
Increase per cm	1.09 (1.06–1.13)	< 0.001	1.09 (1.05–1.13)	< 0.001
Extrahepatic distant metastases				
No	1.00 (reference)		1.00 (reference)	
Yes	1.47 (1.05–2.07)	0.025	1.68 (1.14–2.48)	0.009
Extent of cytoreduction				
Complete cytoreduction	1.00 (reference)		1.00 (reference)	
Incomplete (> 90%)	1.27 (0.92–1.76)	0.15	1.12 (0.76–1.65)	0.56
Incomplete (< 90%)	1.88 (1.01–3.47)	0.045	1.59 (0.77–3.31)	0.21

HR, hazard ratio; CI, confidence interval

^a^Variables with *p* < 0.20 in the univariate analysis were included in the multivariable model. Data are presented after multiple imputation for Ki-67

The results from uni- and multivariable Cox proportional hazards regression analysis of factors associated with progression-free survival from hepatectomy are shown in Table 4. In the univariate analysis, worse progression-free survival was associated with younger age, pancreatic primary tumor, Ki-67 higher than 20%, greater number and size of liver lesions, and incomplete cytoreduction (all *p* < 0.05). In the multivariable analysis, worse progression-free survival remained associated with younger age, pancreatic primary tumor, higher Ki-67, greater number and size of liver lesions, and incomplete cytoreduction (all *p* < 0.05).

**Table 4:** Uni- and multivariable Cox regression analysis of factors associated with progression-free survival from hepatectomy^a^

	Univariate analysis		Multivariable analysis	
	HR (95% CI)	*p* Value	HR (95% CI)	*p* Value
Age at hepatectomy				
Increase per year	0.98 (0.97–0.99)	< 0.001	0.98 (0.98–0.99)	< 0.001
Sex				
Male	1.00 (reference)			
Female	1.01 (0.83–1.22)	0.95		
Primary tumor site				
Small intestine	1.00 (reference)		1.00 (reference)	
Pancreas	1.75 (1.43–2.16)	< 0.001	1.80 (1.40–2.32)	< 0.001
All other sites	1.02 (0.74–1.39)	0.92	1.13 (0.81–1.59)	0.48
Functional tumor				
No	1.00 (reference)			
Yes	1.03 (0.85–1.26)	0.76		
Ki-67 Index (%)				
< 3	1.00 (reference)		1.00 (reference)	
3–20	1.47 (0.99–2.18)	0.06	1.25 (0.83–1.88)	0.28
> 20	2.20 (1.35–3.59)	0.002	1.73 (1.04–2.89)	0.034
No. of metastases				
1–3 Lesions	1.00 (reference)		1.00 (reference)	
4–9 Lesions	1.81 (1.35–2.41)	< 0.001	1.74 (1.29–2.36)	< 0.001
≥ 10 Lesions	1.94 (1.51–2.49)	< 0.001	1.64 (1.23–2.19)	< 0.001
Size of largest metastasis				
Increase per cm	1.04 (1.02–1.07)	< 0.001	1.04 (1.01–1.06)	0.007
Extrahepatic distant metastases				
No	1.00 (reference)		1.00 (reference)	
Yes	1.25 (0.97–1.61)	0.09	1.22 (0.92–1.62)	0.17
Extent of cytoreduction				
Complete cytoreduction	1.00 (reference)		1.00 (reference)	
Incomplete (>90%)	1.78 (1.41–2.25)	< 0.001	1.55 (1.19–2.01)	0.001
Incomplete (90%)	1.78 (1.14–2.78)	0.011	1.51 (0.94–2.41)	0.09

HR, hazard ratio; CI, confidence interval

^a^Variables with *p* < 0.20 in the univariate analysis were included in the multivariable model. Data are presented after multiple imputation for Ki-67

## Discussion

Although cytoreductive hepatectomy has become a widely accepted approach to the management of patients with resectable NET liver metastases because of its potential to prolong survival and manage symptoms, long-term outcomes are poorly characterized. This report describes one of the largest institutional experiences with cytoreductive hepatectomy in the modern era, spanning a period of 21 years. We found that across the entire cohort, overall survival was excellent, with median survival from hepatectomy exceeding 10 years. Additionally, we found that 96% of the patients with functional tumors and symptoms of hormonal excess experienced a marked improvement in symptoms after hepatectomy, with a median symptom-free interval longer than 3 years.

Approximately 37% of our cohort had functional tumors, with carcinoid syndrome as the most common manifestation. Although SSAs can effectively manage symptoms in about 70% of patients with carcinoid syndrome, the majority of patients will have symptomatic progression requiring dose escalation and/or additional treatment.^25–27^ In our cohort, 96% of the patients were rendered asymptomatic or reported a marked improvement in symptoms after hepatectomy, with similar rates across different syndromes. Overall, the response was durable, with a median symptom-free interval of 41 months, which is comparable with prior reports.^9,12^ Although initial rates of symptomatic relief were similar, we found significant differences in the duration of response depending on the extent of cytoreduction, with a median symptom-free interval of 62 months for the patients who underwent complete cytoreduction versus 21 months for those who had gross residual disease. Return of symptoms usually was associated with radiographic progression, suggesting that the same clinicopathologic factors associated with progression-free survival can be used to predict which patients will have the most durable symptomatic response. It should be noted that the reported symptom-free intervals were estimated in the absence of postoperative SSA use, but SSAs often are continued postoperatively in the presence of gross residual disease or restarted at the time of radiographic progression because of their ability to slow disease progression.^28,29^ This may cause the symptom-free interval to appear longer in practice due to masking of recurrent symptoms.

In the current study, the median overall survival from hepatectomy was 135 months for the patients with small intestinal primary tumors, 113 months for those with pancreatic tumors, and 98 months for the patients with other primary tumor sites. These data compare favorably with survival rates for unselected historic controls. For example, in a SEER database analysis from 2008, Yao et al.^2^ reported a median survival of 65 months for patients with metastatic small intestinal NETs and 27 months for those with metastatic pancreatic NETs, although patient selection must be considered because patients selected for surgery may have less extensive disease or other favorable characteristics compared with those managed nonoperatively. Other studies using SEER data have similarly shown shorter overall survival compared with the data presented in this report.^1,4,5^

In the current study, the median progression-free survival from hepatectomy was 23 months for the patients with small intestinal NETs and 10 months for those with pancreatic NETs. This is consistent with prior reports and the notion that cytoreductive hepatectomy is rarely curative but instead may “set back the clock” by delaying hepatic parenchymal replacement with tumor and development of liver failure.^9,13,14^ Survival improved over time, which likely can be attributed to the introduction and advancement of additional therapies, including chemotherapy, embolization techniques, and peptide receptor radionuclide therapy (PRRT).

We found that the strongest predictor of overall survival was Ki-67, with primary tumor site, number and size of hepatic lesions, and the presence of extrahepatic distant metastases also affecting survival. Interestingly, the prevalence of extrahepatic distant metastases increased during the study period, likely due in part to the introduction of more sensitivity staging methods such as DOTATATE positron emission tomography (PET) scans. Patients with poorly differentiated tumors were excluded from this study, but a substantial portion had well-differentiated tumors with Ki-67 higher than 20%, corresponding to grade 3 in the 2017 WHO classification. Cytoreductive hepatectomy in this group has been an area of controversy in the past.^14,15^ However, although patients with a high Ki-67 had markedly worse survival than those with lower values, the median overall survival of 66 months from hepatectomy in this group compares favorably with that for unselected historic control subjects.

In a recent study, Borbon et al.^30^ reported a median overall survival of 19 months for patients with well-differentiated grade 3 gastroenteropancreatic NETs managed nonoperatively, most of whom had distant metastatic disease, and additional studies have shown similar results.^31,32^ Although patient selection must again be considered when outcomes after operative and nonoperative therapy are compared, these results suggest that high grade alone should not preclude patients from consideration for cytoreductive hepatectomy.

Historically, cytoreductive hepatectomy was considered only when at least 90 % of disease could be resected, but recent studies have suggested that lowering this threshold to 70 % may also offer a survival benefit.^33–35^ Due to practice patterns at our institution, the number of patients who underwent less than 90% cytoreduction in the current study was low, representing only about 5% of the cohort. Compared with the patients who underwent complete cytoreduction, those who underwent resection of less than 90% of disease showed significantly worse overall and progression-free survival in the univariate analysis but not in the multivariable analysis, although this analysis likely was underpowered due to the low number of patients who underwent less than 90% cytoreduction.

Although expected fractional tumor reduction has historically been used to determine the feasibility of debulking surgery for individual patients and therefore was used to define the extent of cytoreduction in this study, emerging data suggest that the absolute residual tumor volume may be a better predictor of post-hepatectomy prognosis.^36^ Depending on the initial tumor burden, resection of 90% of disease will result in a wide variation of residual tumor volume, and tumor volume directly correlates with time to progression.^37^ It should therefore be emphasized that the goal should not be simply to resect a certain percentage of disease, but to resect or ablate as much tumor as possible to maximize the potential survival benefit. In future years, volumetric measurements of tumor involvement with calculation of the expected residual tumor volume may play an increasing role in preoperative evaluations and clinical decision-making, as well as post-hepatectomy prognostication for this population.

Long-term survival benefits need to be contrasted with perioperative morbidity and mortality, which overall were low in our study. Major complications were reported in 20% of the patients, and 90-day mortality was 1.6%, similar to prior reports.^38^ As expected, morbidity and mortality rates were higher for the patients who underwent major hepatectomy than for those who underwent minor resections. The use of major hepatectomy decreased during the 21-year study period in favor of parenchyma-sparing resections and intraoperative ablation. Although a subset of patients will continue to require major hepatectomy for adequate cytoreduction, particularly patients who have extensive involvement of one hepatic lobe with relative sparing of the other, parenchyma-sparing procedures should be considered whenever possible to preserve functioning liver parenchyma and decrease the morbidity associated with major resections. This is particularly important considering that recurrence or progression after hepatectomy is almost universal, meaning that most patients will continue to have progressive replacement of liver parenchyma during the remainder of their lives.

Importantly, cytoreductive hepatectomy is only one of many treatment methods that can be used in the management of patients with NET liver metastases. Systemic medical therapy including somatostatin analogs, targeted biologic agents, and cytotoxic chemotherapy, as well as nonoperative liver-directed methods including various ablation and embolization techniques are well-established options that can slow progression and help alleviate symptoms of hormonal excess.^39,40^ Peptide receptor radionuclide therapy (PRRT), recently approved in the United States for the treatment of somatostatin-receptor-positive tumors, has shown very promising effects on progression and survival.^41^ Finally, patients who are not candidates for cytoreductive hepatectomy due to extent or distribution of liver involvement may benefit from liver transplantation, although patient selection is critical.^16^

Our study had several important limitations. First, the retrospective and single-center design indicates that our findings may not be generalizable to all patients. Second, due to the lack of an appropriate comparison group, we are unable to make definitive conclusions about the potential survival benefit of hepatectomy compared with nonoperative therapy. Second, because Ki-67 assessment was rarely performed before the introduction of the 2010 WHO classification for gastrointestinal NETs, this important variable was missing for more than half of the cohort requiring multiple imputation. Third, due to the small number of cases that involved less than 90% cytoreduction, we did not attempt further stratification by extent of cytoreduction for this group, and therefore were unable to compare it with other studies investigating patients who underwent 70–90% cytoreduction. Finally, we were unable to account for other treatment methods because many patients referred to our institution for surgery received the remainder of their care elsewhere, but we recognize that many patients with metastatic NETs are treated with multiple different methods that may affect progression and survival.

In conclusion, our results support the practice of cytoreductive hepatectomy for patients with NET liver metastases when complete or near-complete cytoreduction can be achieved by demonstrating excellent short- and long-term outcomes in terms of both survival and symptomatic relief. Predictors of overall and progression-free survival, including Ki-67, extent of hepatic involvement, and extent of cytoreduction, can be used to inform discussions on prognosis and help guide management decisions.
